# Double-Stranded RNAs in Plant Protection Against Pathogenic Organisms and Viruses in Agriculture

**DOI:** 10.32607/20758251-2019-11-4-13-21

**Published:** 2019

**Authors:** S. Y. Morozov, A. G. Solovyev, N. O. Kalinina, M. E. Taliansky

**Affiliations:** International Laboratory «Resistom», The Skolkovo Innovation Center, Moscow, 143026 Russia**; Belozersky Institute of Physico-Chemical Biology, Lomonosov Moscow State University, Moscow, 119992 Russia; Shemyakin-Ovchinnikov Institute of Bioorganic Chemistry, Russian Academy of Science, Moscow, 117997 Russia

**Keywords:** RNA interference, double-stranded RNA, hairpin RNA, transgenic plants, exogenous dsRNA, regulation of pathogen genes, plant resistance

## Abstract

Recent studies have shown that plants are able to express the artificial genes responsible for the synthesis of double-stranded RNAs (dsRNAs) and hairpin double-stranded RNAs (hpRNAs), as well as uptake and process exogenous dsRNAs and hpRNAs to suppress the gene expression of plant pathogenic viruses, fungi, or insects. Both endogenous and exogenous dsRNAs are processed into small interfering RNAs (siRNAs) that can spread locally and systemically through the plant, enter pathogenic microorganisms, and induce RNA interference-mediated pathogen resistance in plants. There are numerous examples of the development of new biotechnological approaches to plant protection using transgenic plants and exogenous dsRNAs. This review summarizes new data on the use of transgenes and exogenous dsRNAs for the suppression of fungal and insect virulence genes, as well as viruses to increase the resistance of plants to these pathogens. We also analyzed the current ideas about the mechanisms of dsRNA processing and transport in plants.

## INTRODUCTION

RNA interference is an evolutionarily conserved intracellular process that encompasses a dedicated strategy for regulating gene expression. The most important aspect of the RNA interference mechanism is that it does not change the primary chromosome structure of the target genes but is able to significantly attenuate gene expression and lead to a number of changes in the phenotype of cells and whole organisms [1, 2]. The idea of using RNA regions complementary to a specific region of the mRNA of the target gene to suppress the expression of this gene was first described in 1984 [3] as an alternative to classical genetic analysis, i.e., to the generation of mutants that alter the primary structure of the genetic locus. However, the first experiments on the use of antisense RNA to suppress gene activity failed to yield reliably positive results and the mechanisms of this suppression remained poorly understood [4-6]. The term "RNA interference" was first introduced in 1998, when Tabara et al. showed that the process could be initiated by incubation of nematodes in a solution of gene-specific double-stranded RNA fragments [7-9]. However, by that time, explicit indications of the role of complementary RNAs in the regulation of the expression of endogenous eukaryotic genes had already been revealed in transgenic plants and fungi [10-12].

A fundamentally important result was reported in a paper published in 1993 and devoted to the resistance of transgenic tobacco to the tobacco etch potyvirus [13]. A relationship between the detected resistance and RNA interference was proved, because there was co-suppression of both the transgene encoding a viral genome fragment and the virus RNA genome. Therefore, this process should function precisely at the RNA level. During the 1990s, numerous studies reported on RNA interference in many organisms, including fungi, animals, and plants [14, 15]. These studies showed that the RNA interference process is initiated by the Dicer-like enzyme (DCL) that cuts long molecules of viral or cellular double-stranded RNA into short fragments of 21–25 nucleotides, called siRNAs. One of the two chains of each fragment is called a guide strand, because it is further included in the RISC complex. Under the action of this complex, a short single-stranded RNA fragment forms hydrogen bonds with the complementary sequence of an extended RNA molecule and causes cleavage of the latter by a RISC complex protein called Argonaute (AGO). This ensures high specificity of the cleavage. These events lead to the suppression (silencing) of the cell gene or virus replication [1, 16].

The movement of siRNAs in the plant is subdivided into intercellular (local) and systemic (long-distance) transport [17]. This movement occurs through the symplast: i.e., from the place of initiation to neighboring cells through intercellular channels called plasmodesmata, as well as systemically over large distances through conducting tissue of the phloem. Systemic movement of the silencing signal occurs within a few days after initiation and is usually directed from photosynthetic sources (i.e. leaves) to roots and the apical meristem [18, 19]. The systemic silencing signal was identified in plants by direct sampling of the phloem sap [20, 21] and by detection of the signal in grafted parts of the plant [22-24]. Mobile silencing signals include double-stranded siRNA molecules (21–24 nucleotides) [20, 21, 24, 25]. In this case, Dunoyer et al. [26] directly showed that chemically synthesized exogenous, fluorescently labeled siRNAs actually move from cell to cell and over long distances.

Beginning with studies that proved that artificial double-stranded RNAs cause RNA interference [9], the efficiency of this strategy for the protection of plants from pathogenic organisms and viruses has been convincingly proved [27, 28]. In this review, we describe examples of potential practical application of RNA interference in the protection of plants from pathogens.

## EXPRESSION OF DOUBLE-STRANDED RNAs IN TRANSGENIC PLANTS TO SUPPRESS PATHOGENS

**Fig. 1:**
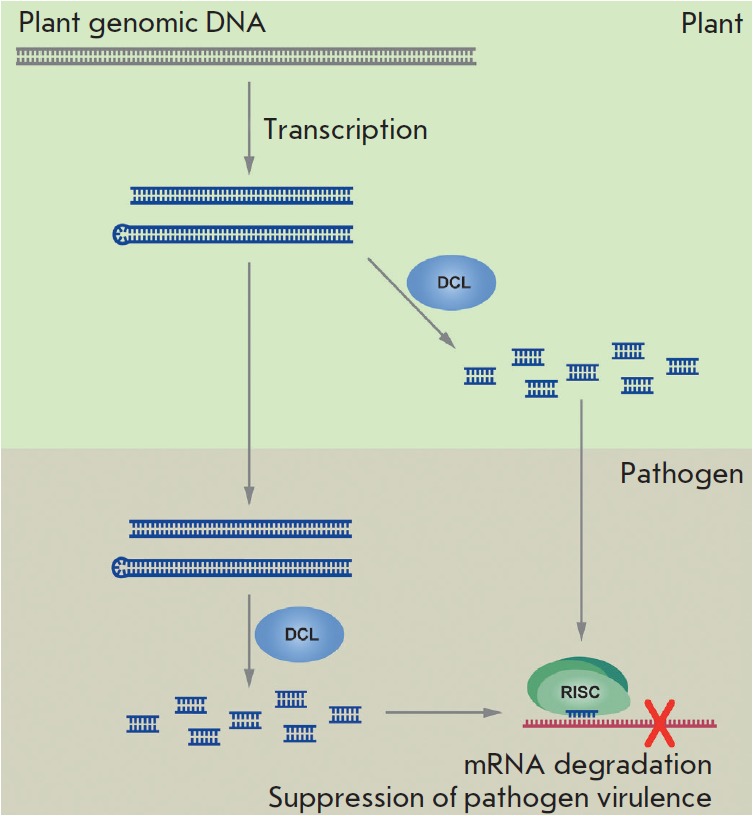
Schematic representation of the use of transgenic dsRNA for RNA interference in plants. Artificial dsRNA is produced from transgenic constructs. Endogenous long dsRNAs are either transported directly into the pathogen’s cytoplasm through an undefined mechanism, or dsRNA molecules (dsRNA or hpRNA) are recognized in the plant by DICER ribonuclease (DCL) that cleaves long dsRNAs into short interfering RNAs. The latter are then transferred to pathogen cells, where they are incorporated into the RNA-induced silence complex (RISC) that directs specific degradation or translational repression of pathogen mRNAs. Interfering RNAs and the RISC complex can form directly in the pathogen cells. Arrows indicate different steps of short interfering RNA induction and dsRNA/siRNA movement between plant cells and phytopathogens

At present, it is obvious that RNA interference may be used to achieve desired pathogen resistance in crop plants by manipulating the expression of the genes of viruses, bacteria, fungi, nematodes, and insects [29, 30]. The method of double-stranded RNA delivery, which was previously widely used for plant protection, is based on the use of transgenic cultures producing pest-specific dsRNAs. The transgene-mediated pathogen suppression method generally involves identification of the pathogen target gene(s) to be suppressed, followed by the generation of a construct producing a hairpin dsRNA, using a genetically engineered cassette containing the target gene (or its fragment) in sense and antisense orientations, as well as a relatively short spacer separating complementary segments, plant transformation, and, finally, screening and evaluation of transformant traits [31, 32] (*Fig. 1*). Transgenic construct-based expression of these dsRNAs in the appropriate host plant often leads to protection against infection. This biotechnological method, called host-induced gene silencing (HIGS), has emerged as a promising alternative to other plant protection methods, because it is highly selective relative to the target organism’s genes. In addition, this method has minimal side effects compared, e.g., with protein-producing transgenes or chemical protective treatment [29, 33].

Over the past 10 years, a number of studies on the use of HIGS to combat fungal diseases have been published [29, 33, 34]. The efficiency of HIGS in fighting against phytopathogenic fungi was proved in an important study published in 2010 [35]. Expression of an interference cassette for the *GUS *marker gene encoding beta-glucuronidase (hairpin (hp)GUS) in tobacco plants was shown to suppress the expression of this gene in *Fusarium verticillioides *fungal cells. However, the efficiency of HIGS against the rust pathogen varied, depending on the gene used. For example, in transgenic wheat plants producing double-stranded RNAs to the *MITOGEN-ACTIVATED PROTEIN KINASE 1 *(*PtMAPK1*), *CYCLOPHILIN *(*PtCYC1*), or *CALCINEURIN B *(*PtCNB*) gene of the rust fungus *Puccinia triticina *[36], disease symptoms decreased by 51–68% and fungus biomass dropped by 59–69% compared with control vector constructs. In wheat leaves expressing these constructs, symptoms of *Puccinia graminis *infection also decreased slightly. Therefore, meticulous selection of the target genes may obviously enhance the efficiency of HIGS and provide a wider range of resistance to rust fungi.

An obvious effect of HIGS was also demonstrated in cereals infected with the powdery mildew pathogen *Blumeria graminis *[37]. A reduction in powdery mildew symptoms and a decrease in the number of functional haustoria inside epidermal cells were found in barley or wheat plants with HIGS-mediated suppression of the Avra10 effector gene. Suppression of fatty acid metabolism genes using the HIGS strategy has revealed the efficiency of this method in generating disease tolerance in some other crop plants. HIGS-mediated suppression of a rice gene, *OsSSI2, *led to enhanced resistance to the fungus *Magnaporthe grisea *and the leaf blight bacterium *Xanthomonas oryzae *[38]. Enhanced resistance against *M. grisea *in rice plants was achieved through the suppression of two genes: namely, *OsFAD7 *and *OsFAD8*, which encode Ω-3 fatty acid desaturase [39]. Furthermore, suppression of the genes involved in lignin production led to enhanced resistance of soybean plants to the phytopathogen *Sclerotinia sclerotiorum *[40].

In contrast to the presented data, HIGS-mediated silencing of the genes of the oomycete *Phytophtora parasitica *failed to initiate an obvious protective response in transgenic arabidopsis plants expressing *PnPMA1 *dsRNA [41]. However, other examples indicate the possibility of a successful use of HIGS against oomycetes. For example, transgenic tobacco plants expressing glutathione S-transferase gene dsRNA developed noticeable resistance to a tobacco phytophthora strain [42].

The problems related to using transgenic plants expressing dsRNAs to the genes of parasitic nematodes are summarized by Lilley et al. [43]. They noted that complex relationships between the plant and the parasite remain not fully understood. In particular, the inability to transform parasitic nematodes and generate their mutant lines obstructs our understanding of gene functions, which, in turn, complicates the identification of genes that may be effective targets for RNA interference. However, data for other cellular pathogens, in particular the soybean nematode *Heterodera glycines *and fungi, can be used for this purpose. For example, Youssef et al. [44] used the HIGS strategy to suppress the *HgALD *gene (fructose-1,6-diphosphate-aldolase), which reduced the number of female offspring by 58%.

Silencing of the housekeeping genes of the root nematode by the expression of dsRNA in the host plant also enhanced anti-nematode resistance [45]. Ibragim et al. [46] were able to successfully reduce the formation of *Meloidogyne incognita *galls in soybean roots by suppressing the genes encoding tyrosine phosphatase and fructose-1,6-diphosphate aldolase, a key glucogenesis enzyme.

An alternative HIGS strategy, which is aimed at combating nematodes, involves the genes necessary for parasitism [47, 48]. Genes *flp-14 *and *flp-18 *of the gall nematode *M. incognita *encode neuropeptides that are involved in nematode migration and host root invasion [47]. HIGS-mediated silencing of either of the two genes in transgenic tobacco plants reduces the infection of most lines with this nematode. Fertility of females decreases by ~50–80%. Parasitism can also be disrupted by HIGS-mediated silencing of the genes encoding the nematode effector proteins that play an important role in establishing successful parasitic relationships with the host. Transgenic *Arabidopsis thaliana *plants expressing dsRNAs for regions of the conserved root-knot nematode effector gene, *16D10*, encoding a small secretory peptide assisting in the selection of feeding sites develops a wide spectrum of resistance to *M. incognita *[48]. Reduced susceptibility to *M. incognita *was also detected in the roots of transgenic grape plants expressing constructs based on a hairpin of a *16D10 *gene sequence fragment [49]. Sindhu et al. [50] used suppression of four different genes involved in the parasitism of the sugar beet nematode (*Heterodera schachtii*) in the *A. thaliana *host expressing dsRNAs. Although total resistance was not achieved, the number of mature female nematodes decreased to 23–64% in different transgenic plant lines.

RNA interference is also used to control insect pests causing significant crop losses [51-53]. Mao et al. [54] developed a strategy that controls an insect’s sensitivity to plant phytotoxins. After an insect attack, plants synthesize a variety of secondary metabolites aimed at reducing the viability of pests. In response, some insects have developed the ability to detoxify these compounds, which is often associated with the activity of cytochrome P450 monooxygenase. According to a genetic and biochemical analysis, expression of cytochrome P450 (*CYP6AE14*) in cotton worm larvae (*Helicoverpa armigera*) is necessary to initiate resistance to gossypol, a cotton phytotoxin [54]. Furthermore, expression of CYP6AE14 dsRNA in larvae grown on transgenic arabidopsis, tobacco, or cotton plants reduces synthesis of the appropriate protein and enhances sensitivity to gossypol [54, 55]. Later, the same authors showed that the protection level may be increased by co-expression of CYP6AE14 dsRNA and cysteine protease [56]. The host-plant-induced dsRNA causing cytochrome P450 silencing was also used to enhance sensitivity to deltamethrin pyrethroid that is used to control cotton pests [57]. These results suggest that cytochrome P450-targeted enzymatic systems are an effective pathway to reducing resistance to pyrethroids.

Crop plants encoding heterologous proteins or overexpressing these proteins are fundamentally different from crops that encode cassettes for the synthesis of interfering dsRNAs. RNAs are known to be non-toxic to humans, while foreign proteins produced by transgenic plants can, in some cases, be toxic or allergenic [58]. Therefore, transgenic crops with RNA-based resistance genes are much safer for humans than crops with excessive expression of proteins and do not require a determination of acute oral toxicity and assessment of the digestibility of an administered RNA component. Some biosafety problems are associated with the use of transgenic plants expressing dsRNAs, because transcriptional gene silencing by chromatin modification can lead to hereditary changes that have an adverse effect. This fuels public concern about the safety of genetically modified organisms [59]. Furthermore, many countries have put legislative restrictions on the cultivation of transgenic plants (Law Library of Congress (US): Global Legal Research Directorate; Restrictions on Genetically Modified Organisms; Global Legal Research Center: Washington, DC, USA, 2014, p. 242). Therefore, the development of new, environmentally friendly approaches to enhancing pest resistance without significant modifications in the plant genome is an important undertaking. One of these approaches is genome editing using the CRISPR/Cas system. First, CRISPR/Cas systems can be used to introduce point mutations or small deletions into specific genes of the host plants in order to block the mechanisms promoting the spread of the pathogen in the plant. Second, CRISPR/Cas systems can be developed for the mutagenesis of pathogen genomes. For example, CRISPR/ Cas9 systems can be targeted directly at DNA- or RNA-containing viruses [60].

## METHODS FOR DELIVERY OF ARTIFICIAL DOUBLE-STRANDED RNAs IN PLANTS: DIRECT TREATMENT OF PLANTS WITH DSRNA

RNA interference-based methods have been proved to be an effective strategy for protecting plants from the diseases caused by viral and cellular pathogens. However, the possibility of a widespread use of HIGS remains very doubtful because the development of genetically modified crop plants takes a lot of time and is still widely mistrusted by people in many European countries.

The search for alternative strategies was facilitated by the results of earlier studies, which showed that dsRNA solutions may be used for RNA interference of the nematode *Caenorhabditis elegans *[7]. Furthermore, successful experiments on the suppression of the growth and reproduction of parasitic plant nematodes *in planta *proved that RNA interference in this case may be a promising method for reducing the viability of pests [43]. At the moment, these studies are innovative and may lead to significant progress in the development of an RNA interference-based approach to plant protection by direct introduction of exogenous dsRNA complementary to the pathogen genome. These studies will clarify the following important issues: (i) the methods and mechanistic basis for the introduction of dsRNAs into plants; (ii) solving the problems of transport, processing, and stability of dsRNAs in the external environment and in cells; and (iii) implementation of large-scale production and purification of exogenous dsRNA to make this approach economically viable. Several alternative methods for dsRNA delivery, which do not involve plant transformation, have been proposed. In particular, dsRNA can be translocated into the plant vascular system (xylem and phloem) through roots or by direct injection of RNA molecules into a tree trunk [28, 61-66].

**Fig. 2:**
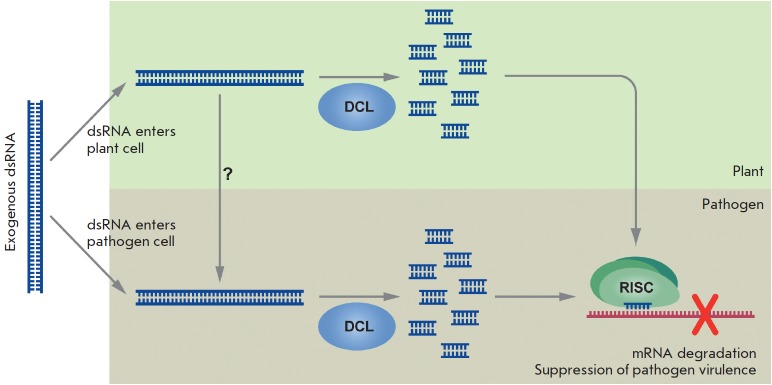
Schematic representation of the use of exogenous dsRNA for RNA interference induction and degradation of target plant pathogen mRNAs. Exogenous artificial dsRNA is dissolved and applied to plant leaves, flower buds, roots, or seeds. Uptake and transport of exogenous dsRNAs occur through an undefined mechanism. dsRNA or hpRNA molecules are recognized by DICER-like (DCL) ribonuclease that cleaves long dsRNAs into siRNAs. siRNAs are then incorporated into the RNA-induced silencing complex (RISC) that guides sequence-specific degradation or translational repression of homologous pathogen mRNAs. Arrows depict different steps of the RNAi induction process and dsRNA/ siRNA movement between plant cells and plant pathogens

However, the spraying of plants (mainly leaves) is currently considered the most promising method. This method is called spray-induced gene silencing (SIGS). Exogenous interfering dsRNAs can either be directly uptaken by pest cells or transferred first to plant cells and then to pathogen cells (*Fig. 2*) [64, 67, 68]. In this regard, it is important to note that locally sprayed RNAs also inhibit pathogen virulence in distal untreated leaves [68, 69]. Obviously, these dsRNAs, or shorter products of their processing, are capable of systemic spread in plants.

Initial "naked" dsRNA preparations have been shown to protect plants from microbial pathogens for 10 days after spraying [64, 67, 68]. However, incorporation of dsRNAs into hydroxide nanolayers, called BioClay, was recently shown to increase the duration of the protection against infection by more than 20 days [69]. BioClay nanolayers prevented degradation of dsRNA by RNase or sunlight. Because these nanoparticles and incorporated RNA are non-toxic and easily decomposed, this method is considered environmentally friendly. Moreover, it increases the efficiency of SIGS in combating plant diseases in the field [70]. Thus, advances in nanoparticle technology have markedly improved the potential efficiency of SIGS for plant protection. In addition, chitosan polymers were also used to encapsulate dsRNA and achieve RNA interference. Chitosan nanoparticles were produced by self-assembly of the polymer with dsRNA using electrostatic interactions between the positive and negative charges of amino groups in the chitosan and phosphate groups in nucleic acid, respectively. This method is well-suited for long dsRNAs. Chitosan nanoparticles, when applied to plants, can enter a pest’s body, along with food. This system is very inexpensive and highly efficient. In addition, chitosan polymers are non-toxic and readily biodegradable [63, 69, 71, 72].

The length of exogenous dsRNA is very important for an efficient suppression of the genes of plant pathogens. The dsRNA length required to achieve a pronounced effect varies depending on the pathogen type and taxon. Insect studies have shown that the dsRNA length required for successful RNA interference ranges from 140 to 500 nucleotides in most cases. For viruses, this length is more than 200–300 nucleotides [28]. In general, each particular gene is believed to require screening for several dsRNA types of different lengths and locations. In addition, dsRNA can be either very specific to the target gene of a particular pathogen type or designed for a wider range of closely related species [69, 71, 72].

The efficiency of RNA interference induction by exogenous dsRNAs also depends on their optimal (sufficiently high) concentration, which in practical applications requires the production of large amounts of dsRNA [73-75]. In RNA interference experiments, dsRNAs have been produced *in vitro *by bidirectional transcription using T7 polymerase [76, 77]. However, it is obvious that such a system is unsuitable for large-scale production, for economic reasons. Therefore, it has been proposed to use an inducible cassette with the T7 phage RNA polymerase promoter, which expresses dsRNA in the RNase III-deficient *Escherichia coli *strain HT115, M–JM109, or M–JM109lacY [78-81]. In addition, stable and efficient systems for dsRNA production in *Pseudomonas syringae *bacteria [82] and *Saccharomyces cerevisiae *yeast [83] were recently developed. Obviously, the listed microbiological expression systems can potentially be used for large-scale and inexpensive production of dsRNAs for practical applications of SIGS in agriculture.

In recent years, the SIGS system has been shown to be effectively usable to control plant pathogenic fungi. Application of dsRNAs, which were synthesized *in vitro *and directed against a number of fungal genes, to the leaf surface was found to reduce the spread of infection by blocking growth, altering morphology, and reducing pathogenicity and lead to less pronounced manifestations of the disease [67, 68, 84, 85]. The use of exogenous dsRNA on plant surfaces is currently regarded as an innovative strategy for protecting plants from fungal infection [28, 63, 64, 67]. It is supposed that there may be two ways for dsRNAs deposited on the plant surface to occur in fungal cells: (i) after the spraying of plants, dsRNAs immediately penetrate fungal cells and undergo processing into siRNAs; and (ii) RNAs enter plant cells and form short siRNAs that are translocated to fungal cells (*Fig. 2*) [67, 68, 86]. The effect of *Myo5 *gene-silencing in *Fusarium asiaticum *is found to linger only if dsRNA continuously enters fungal cells, because *F. asiaticum *cannot support the amplification of secondary siRNAs. The findings of Song et al. [86] indicate that dsRNAs entering plants are processed into siRNAs that are then amplified by plant RNA-dependent RNA polymerase (RdRP), resulting in the formation of secondary siRNAs. Interestingly, uptake of dsRNA through the wound surface of tip cut wheat coleoptiles was more efficient than through the intact surface. In addition, penetration of dsRNA was enhanced by a nonionic surfactant, Silwet L-77 [84, 86].

Over the past few years, numerous studies have shown that dsRNAs that are complementary to a number of important insect pest genes can become an effective inducer of SIGS and increase insect mortality, decrease their growth rate and fertility, and reduce their sensitivity to insecticides [87]. Treatment of leaves with artificially synthesized dsRNAs targeted at the genes involved in insect development significantly increases mortality and inhibits insect growth [88-92]. This effect can be achieved by irrigating the roots of plants by dsRNA, which leads to effective suppression of the target gene and abnormal development of insect pests [74, 76, 93]. RNA interference using exogenous dsRNAs can be used for a very wide range of insect genes. For example, suppression of the expression of two ATPase genes in *Diabrotica undecimpunctata *and *Leptinotarsa decemlineata *reduced insect survival by 40–50% [76]. Mortality of the cabbage moth (*Plutella xylostella*) on leaves sprayed with dsRNAs to *Pl. xylostella *acetylcholinesterase genes, *AChE1 *and *AChE2*, reached 74 and 89%, respectively [94]. In addition, SIGS to the juvenile hormone acid O-methyltransferase (JHAMT) and vitellogenin genes significantly reduced the levels of these proteins (up to 85–90%) in several taxonomically distant insects [95].

At the moment, the subtle mechanisms of dsRNA penetration from plants into pest cells are not fully understood. Obviously, dsRNA directly penetrates fungal hyphae from plant cells and intercellular spaces. The mechanisms of dsRNA action on nematodes and insects are less understood. The natural pathway is initial RNA penetration from ingested plant sap into digestive tract cells. In this case, endocytosis probably plays an important role. For example, two genes required for efficient penetration of dsRNA during nutrition were found in nematodes. They were *Systemic RNAi-deficient *(*SID*) genes [64, 67]. The *SID-2 *gene encodes a transmembrane protein involved in a rather slow uptake of dsRNA by endocytosis, while the *SID-1 *gene product is necessary for fast, not related to endocytosis, transport and forms channels in the plasma membrane [64, 67].

The effect of exogenous dsRNAs on virus resistance in various species, including tobacco, tomato, corn, papaya, and orchids, has been analyzed in several experimental studies. In this case, plants were treated either with RNA synthesized *in vitro *or with nucleic acid preparations purified from bacterial strains expressing dsRNA or hpRNA [79, 80, 96, 97]. dsRNAs targeted at virus replicase or coat protein genes was found to delay the development of disease, reduce infection symptoms and the number of infected plants, and decrease the virus titer [28, 65]. In addition, it was confirmed that the protective effect induced by dsRNA or hpRNA lingers for at least 20–70 days after inoculation of the virus [72, 98].

In conclusion, it should be noted that SIGS is a targeted and environmentally friendly strategy for plant protection both after and before harvest and, obviously, minimally harmful to the health of consumers. In addition, because the highly conservative pathogen genes necessary for their growth or virulence are often selected for SIGS, pathogens are not able to generate a sufficient amount of mutations in these important genes to avoid the influence of SIGS and simultaneously preserve their vital functions. Finally, it should be emphasized once again that the SIGS technology is much more acceptable to the public than, e.g., chemical treatments, and its development requires significantly less time than the creation of stable transgenic cultures.

## Abbreviations

RNA: ribonucleic acid
RISC: RNA-induced silencing complex
siPHK: small interfering RNA
dsRNA: double-stranded RNA
hpRNA: hairpin RNA
HIGS: host-induced gene silencing
SIGS: spray-induced gene silencing
